# Supplementary material for: Dynamic transcriptomic profiles of zebrafish gills in response to zinc supplementation
Source: BMC Genomics. 2010 Oct 11;11:553. doi: 10.1186/1471-2164-11-553 (PMC3091702; doi:10.1186/1471-2164-11-553)
Supplement: Additional file 2 — Interactive Direct Interaction Network representing the molecular interactions between zinc, copper, iron, calcium and proteins encoded by transcripts changed by zinc supplementation. Mini web-site containing index.html and hyperlinked pages in subdirectory describing a Direct Interaction Network automatically generated based on curated interactions contained within the proprietary PathwayArchitect database. Ovals represent proteins and the circles symbolize metal ions. Objects are coloured by their abundance in zebrafish at the time-point they were significantly different from the control is a scale from -4 fold (dark green) to +4 fold (dark red). Where significant differences were found at more than one time-point, the colour overlay shows expression at the first instance. Dark blue squares denote 'binding', and light blue squares 'expression'; green squares stand for 'regulation', green diamonds for 'metabolism', and green circles for 'promoter binding'. Arrow heads indicate directionality of the interaction where annotated. All nodes and edges can be further interrogated by selecting the relative area of the image. [file 1471-2164-11-553-S2.zip › PathwayArchitect Zn xs DIN/408180.html]

# PROTEIN MODIFICATION:

|  |  |
| --- | --- |
| Type | PROTEIN MODIFICATION |
| Effect | None |


---

|  |  |
| --- | --- |
| Score | 0 |


---

|  |  |
| --- | --- |
| Reference Count | 9 |


---

|  |  |
| --- | --- |
| Mechanism | Unknown |


---

|  |  |
| --- | --- |
| Reference:0 || Sentence | "In the absence of external Ca2+, phenylephrine induced a small transient increase in [Ca2+]i, MLC phosphorylation and generation of force." |
| PMID | 8857588 |
| Year | 1996 |
| Species | Rat |
| Journal | Naunyn Schmiedebergs Arch Pharmacol |
| RefScore | 1 |
| Source | PArchNLP |
  |
|  |  |


---

|  |  |
| --- | --- |
 Reference:1 || Sentence | "To explore the possibility that intracellular Ca(2+) mobilization by oxytocin may also contribute to MLC phosphorylation, we investigated the relative contributions of these Ca(2+) sources to oxytocin signal transduction in myometrium of pregnant rat." |
| PMID | 11757073 |
| Year | 2001 |
| Species | Rat |
| Journal | J Mol Recognit |
| RefScore | 1 |
| Source | PArchNLP |
  ||  |  |


---

|  |  |
| --- | --- |
 Reference:2 || Sentence | "Our results clearly indicate that extracellular Ca(2+) influx, rather than release from Ca(2+) storage sites, is essential for oxytocin-induced MLC phosphorylation." |
| PMID | 11757073 |
| Year | 2001 |
| Species | Rat |
| Journal | J Mol Recognit |
| RefScore | 1 |
| Source | PArchNLP |
  ||  |  |


---

|  |  |
| --- | --- |
 Reference:3 || Sentence | "The third stage is initiated by an abrupt phosphoinositide breakdown via the synergistic action of TXA2 and occupation of the collagen receptor, and the resulting inositol trisphosphate may induce the second-phase Ca2+ mobilization to produce the second-phase MLC phosphorylation together with aggregation and secretion." |
| PMID | 3115969 |
| Year | 1987 |
| Species | Rat |
| Journal | J Biochem (Tokyo) |
| RefScore | 1 |
| Source | PArchNLP |
  ||  |  |


---

|  |  |
| --- | --- |
 Reference:4 || Sentence | "Free Ca2+ increased MLC peptide phosphorylation by beta-cell extracts in vitro." |
| PMID | 9357809 |
| Year | 1997 |
| Species | Rat |
| Journal | Am J Physiol |
| RefScore | 1 |
| Source | PArchNLP |
  ||  |  |


---

|  |  |
| --- | --- |
 Reference:5 || Sentence | "CONCLUSIONS: The increased Ca(2+)-responsiveness of the contractile apparatus in end-stage failing human hearts cannot be explained by a shift in contractile protein isoforms, but results from the complex interplay between changes in the phosphorylation status of MLC-2 and TnI." |
| PMID | 12504812 |
| Year | 2002 |
| Species | Human |
| Journal | Cardiovasc Res |
| RefScore | 2 |
| Source | PArchNLP |
  ||  |  |


---

|  |  |
| --- | --- |
 Reference:6 || Sentence | "These data suggest that ANP may not act at site of either agonist-induced membrane PI hydrolysis or intracellular Ca2+-signal system, but may partly be involved in phosphorylation/dephosphorylation of 20 K-Da MLC in cultured rat VSMC." |
| PMID | 2853774 |
| Year | 1988 |
| Species | Rat |
| Journal | Jpn Circ J |
| RefScore | 1 |
| Source | PArchNLP |
  ||  |  |


---

|  |  |
| --- | --- |
 Reference:7 || Sentence | "Physiol. 271 (Cell Physiol. 40): C242-C247, 1996]. We hypothesized that Ca2+ inhibits renin secretion, via phosphorylation of 20-kDa myosin light chain (MLC20), by activating MLCK. In the present studies, we have investigated the types of protein phosphatase (PP) involved in the control of renin secretion through inhibition of MLC dephosphorylation using inhibitors of various types of serine/threonine-specific protein phosphatases." |
| PMID | 9815125 |
| Year | 1998 |
| Species | Rat |
| Journal | Am J Physiol |
| RefScore | 1 |
| Source | PArchNLP |
  ||  |  |


---

|  |  |
| --- | --- |
 Reference:8 || Sentence | "An imposed ionomycin-triggered cystolic-free Ca(2+) concentration ([Ca(2+)](c)) elevation restored the expression, phosphorylation, and insertion of MLC2v into sarcomeric structures and in turn the myofibrillogenesis." |
| PMID | 12105184 |
| Year | 2002 |
| Species | Mouse |
| Journal | J Cell Biol |
| RefScore | 1 |
| Source | PArchNLP |
  |


---

|  |  |
| --- | --- |
